# Electrical storm in an acquired short QT syndrome successfully treated with quinidine

**DOI:** 10.1002/ccr3.2282

**Published:** 2019-06-23

**Authors:** Michael Spartalis, Efthimios Livanis, Eleftherios Spartalis, Alexandros Tsoutsinos

**Affiliations:** ^1^ Division of Cardiology Onassis Cardiac Surgery Center Athens Greece; ^2^ Laboratory of Experimental Surgery and Surgical Research, Medical School University of Athens Athens Greece; ^3^ Department of Pediatric Cardiology & Adult Congenital Heart Disease Onassis Cardiac Surgery Center Athens Greece

**Keywords:** arrhythmia, electrical storm, quinidine, short QT

## Abstract

Short QT syndrome (SQTS) is a malignant heart disorder defined by the presence of ventricular arrhythmias causing syncope and sudden cardiac arrest. The prevalence in the pediatric population is 0.05%. Quinidine is an established agent for pharmacological prophylaxis in SQTS patients, but can also terminate an electrical storm.

## 1 CASE PRESENTATION

A 14‐year‐old male with no past medical history presented to our emergency department after an episode of loss of consciousness in the classroom. Physical examination was unremarkable.

**Figure 1:**
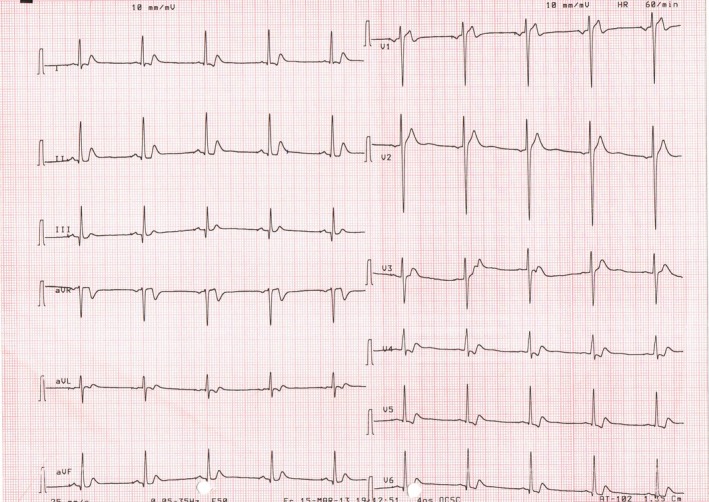
The 12‐lead surface electrocardiogram (ECG) obtained on the admission showed sinus rhythm with a heart rate of 60 bpm, a QT of 320 ms and a QTc of 320 ms

The electrocardiogram showed a QT of 320 ms and a QTc of 320 ms (Figure 1). Echocardiography, cardiac magnetic resonance imaging, and coronary angiography were normal. The patient went on to have a positive programmed ventricular stimulation for inducible ventricular tachycardia and underwent an implantable cardioverter defibrillator implantation. Genetic testing did not detect any mutations. A short QT interval is usually considered if QTc is ≤340 ms,^1^, ^2^ and a diagnosis of SQTS was established.

**Figure 2:**
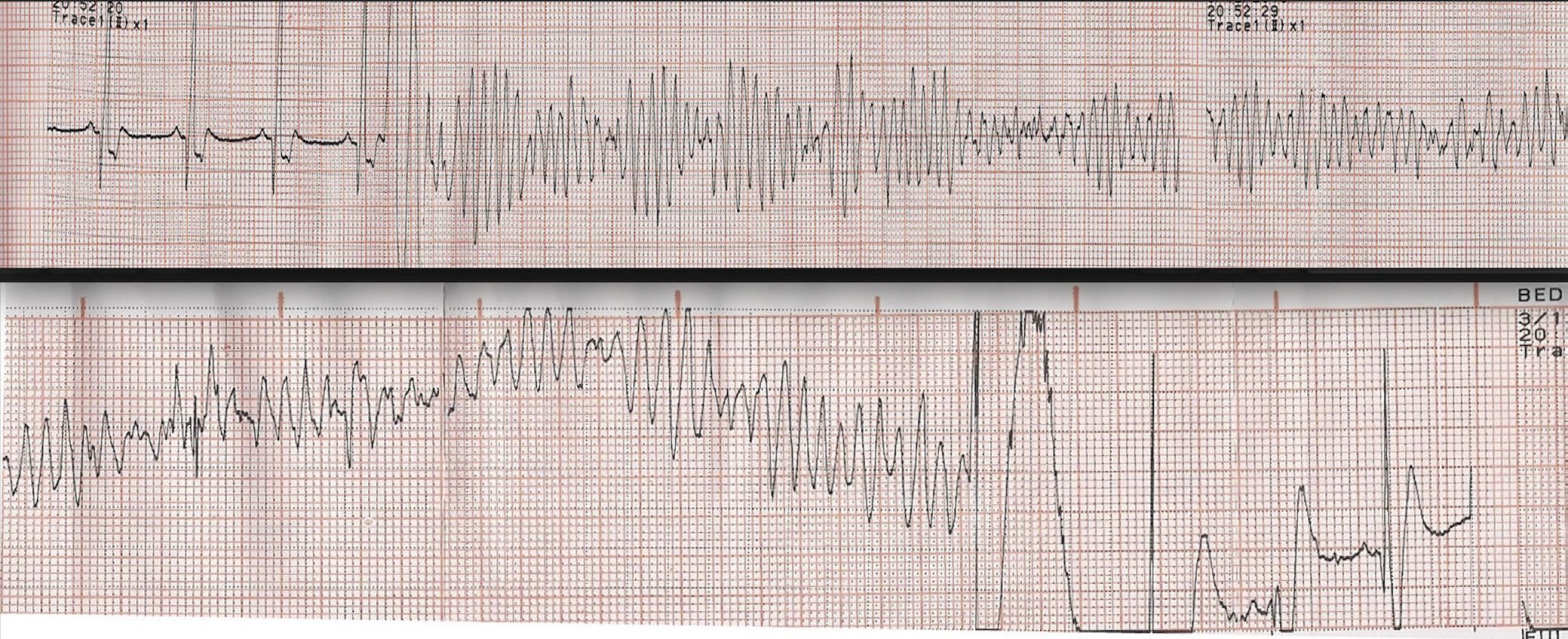
ECG strip showing an episode of Torsade de pointes degenerate into ventricular fibrillation. The episode was induced after a premature ventricular beat due to R/T phenomenon

After 5 months, the patient presented with electrical storm with 16 successive episodes of Torsade de pointes that degenerate to ventricular fibrillation (Figure 2). The episodes were induced by a premature ventricular beat due to R/T phenomenon. Esmolol and amiodarone intravenous infusion were proven ineffective, as well as sotalol therapy. The patient received a 500 mg oral loading dose of quinidine and then 250 mg two times daily. Quinidine led to the rapid disappearance of premature beats and arrhythmia episodes.

Quinidine can normalize the QT interval, leading to significant QT prolongation, a longer ventricular effective refractory period, and is an established preventive therapy for SQTS.^1^, ^2^

To the best of our knowledge, only a few cases of electrical storm in SQTS have been described in the pediatric population, and this is the first case demonstrating the beneficial effect of quinidine in the suppression of electrical storm in SQTS patients.^1^, ^2^

## CONFLICT OF INTEREST

The authors report no financial relationships or conflicts of interest regarding the content herein.

## AUTHOR CONTRIBUTIONS

MS, AT: Conception and design of the research and writing of the manuscript. AT, MS: Acquisition of data. ES, MS: Analysis and interpretation of the data. EL: Critical revision of the manuscript for intellectual content.
